# Are perceived barriers to accessing health care associated with inadequate antenatal care visits among women of reproductive age in Rwanda?

**DOI:** 10.1186/s12884-020-2775-8

**Published:** 2020-02-10

**Authors:** Marie Paul Nisingizwe, Germaine Tuyisenge, Celestin Hategeka, Mohammad Ehsanul Karim

**Affiliations:** 1 University of British Columbia, Vancouver, Canada; 2 Simon Fraser University, Burnaby, Canada; 3 Centre for Health Evaluation and Outcome Sciences, St. Paul’s Hospital, Vancouver, Canada

**Keywords:** Antenatal care, Barriers to care, Maternal health, Demographics and health survey, Rwanda

## Abstract

**Background:**

Maternal and child mortality remain a global health concern despite different interventions that have been implemented to address this issue. Adequate antenatal care (ANC) is crucial in reducing maternal and neonatal morbidity and mortality. However, in Rwanda, there is still suboptimal utilization of ANC services. This study aims to assess the relationship between perceived barriers to accessing health care and inadequate ANC visits among women of reproductive age in Rwanda.

**Methods:**

This study is cross-sectional using secondary data from the 2014–15 Rwanda demographic and health survey (RDHS). The study included 5876 women aged 15–49 years, and the primary outcome of the investigation was inadequate ANC visits defined as delayed first ANC visit and non-completion of at least four recommended visits during the pregnancy period. The primary exposure was perceived barriers to accessing health care, operationalized using the following 4 variables: distance to the health facility, getting money for treatment, not wanting to go alone and getting permission to go for treatment. A survey-weighted multivariable logistic regression analysis and backward elimination method based on Akaike information criterion (AIC) was used to select the final model. We conducted a number of sensitivity analyses using stratified and weighting propensity score methods and investigated the relationship between the outcome and each barrier to care separately.

**Results:**

Of 5, 876 women included in the analysis, 53% (3132) aged 20 to 34 years, and 44% (2640) were in the lowest wealth index. Overall, 64% (2375) of women who perceived to have barriers to health care had inadequate ANC visits. In multivariable analysis, women who perceived to have barriers to health care had higher odds of having inadequate ANC visits (OR: 1.14; 95% CI: 0.99, 1.31). However, the association was borderline statistically significant. The findings from sensitivity analyses were consistent with the main analysis results.

**Conclusion:**

The study suggests a positive association between perceived barriers to health care access and inadequate ANC visits. The findings speak to a need for interventions that focus on improving access to health care in Rwanda to increase uptake of ANC services.

## Background

Maternal and neonatal mortality remain a global health concern despite different interventions that have been implemented to address this issue [1]. Sub-Saharan Africa (SSA) is by far among the regions with the highest ratios of maternal mortality with 351 per 100 000 live births and high neonatal mortality rates with 20 per 1000 live births [2]. Several studies have shown that 15% of maternal and newborn deaths in SSA are attributed to pregnancy complications that are a result of inadequate pregnancy follow-up [2–4].

Timely and frequency of ANC are crucial in reducing delivery complications, maternal and neonatal mortality [5, 6]. A study conducted in Zimbabwe found a 42 and 29% decrease in neonatal and under-five mortality respectively as a result of utilization and quality improvement of ANC services [7]. Before 2016, the World Health Organization (WHO) ANC guidelines—also known as Focused Antenatal Care (FANC)—recommended at least four ANC visits during the time of the pregnancy. The guidelines recommended the first ANC visit to take place within 3 months of pregnancy (timely ANC) and subsequent visits in 24–26 weeks, 32 weeks and 36–38 weeks [5, 8]. The new WHO ANC guidelines recommend at least eight ANC visits including one visit in the first trimester, two visits in the second trimester and five visits in the third trimester [9]. This study used the former guidelines since DHS was conducted before the development of the new guidelines. WHO developed these guidelines to improve ANC in developing countries; however, the available evidence shows poor utilization of ANC in low-and-middle income countries [8, 10–12].

Rwanda is among the few countries that achieved maternal and child millennium development goals (MDGs) with a child mortality decrease of more than 70% comparing 2002 to 2015 [13, 14] and a maternal mortality ratio decreased from 1020 deaths per 100,000 live births in 2000 to 290 deaths per 100,000 live births in 2015 [15]. However, the country’ maternal and neonatal death rates remains high and had slower decline compared to the post-neonatal mortality in the MDGs era. Therefore, there is a need for combined efforts towards the progress to achieving sustainable development goals on maternal and neonatal health [13]. Among these efforts, there is a call to promoting the use of ANC services among women in Rwanda as research shows that there is still suboptimal utilization of ANC services. Based on recent findings, 64% of women did not complete at least four ANC visits, and only 56% of these women had a timely first ANC visit [11]. Further, 59% of women reported at least one barrier to accessing health care [11, 16]. Limited access to health care leads to an increased risk of poor health outcomes [17]. A study conducted in rural Tanzania found a strong association between distance to the health facility and maternal mortality [18], hence speaking to physical access barriers. Other studies have reported treatment cost and demand at work to be potential barriers of health services utilization [19], the former being related to financial access and the latter being availability and accommodation of women’s needs while seeking maternal health services [20].

To date, there is a paucity of studies that assessed the relationship between perceived barriers to health care and inadequate ANC visits in Rwanda. A study conducted in Rwanda assessed the determinants of timing of first ANC visit [21]. This study considered only distance to a health facility as a barrier to health care; the authors did not consider other substantial barriers to care such as treatment cost, time and ability to go alone to a health facility [16, 22]. Further, the study did not investigate subsequent ANC visits [5] and recommended future research in this area [21]. Another study that assessed these barriers and frequency of ANC visits in Rwanda was conducted only in two out of five provinces of Rwanda and included only women who had a child in the past 13 months [23] which limits the generalizability of the findings. The recent 2015 DHS data gives an opportunity to assess whether there is an underlying relationship between perceived barriers to health care and ANC services utilization using a country representative sample. Therefore, this study aims to investigate the relationship between perceived barriers to health care and inadequate ANC visits among women of reproductive health in Rwanda after adjusting for potential confounders.

We hypothesized that women who perceived to have barriers to health care are more likely to have inadequate ANC visits. This study will contribute to future interventions that are essential in improving ANC utilization and access to health care in Rwanda.

## Methods

### Study design and data source

This was a cross-sectional study using secondary data from the 2014–15 Rwanda DHS. This nationally representative survey included individuals aged 15 years and older living in 30 districts of Rwanda. A representative sample of households was selected using two-stage cluster sampling. At the first stage, 492 villages were selected, and 12,792 households were randomly sampled within these villages at the second stage [11]. Sample weights were available in the data. The overall response rate at the household level was 99.9% (12,699 households were interviewed) and 99.5% at the individual level. More details on sampling and data collection procedures are discussed elsewhere [11].

### Analytic sample

**Fig. 1:**
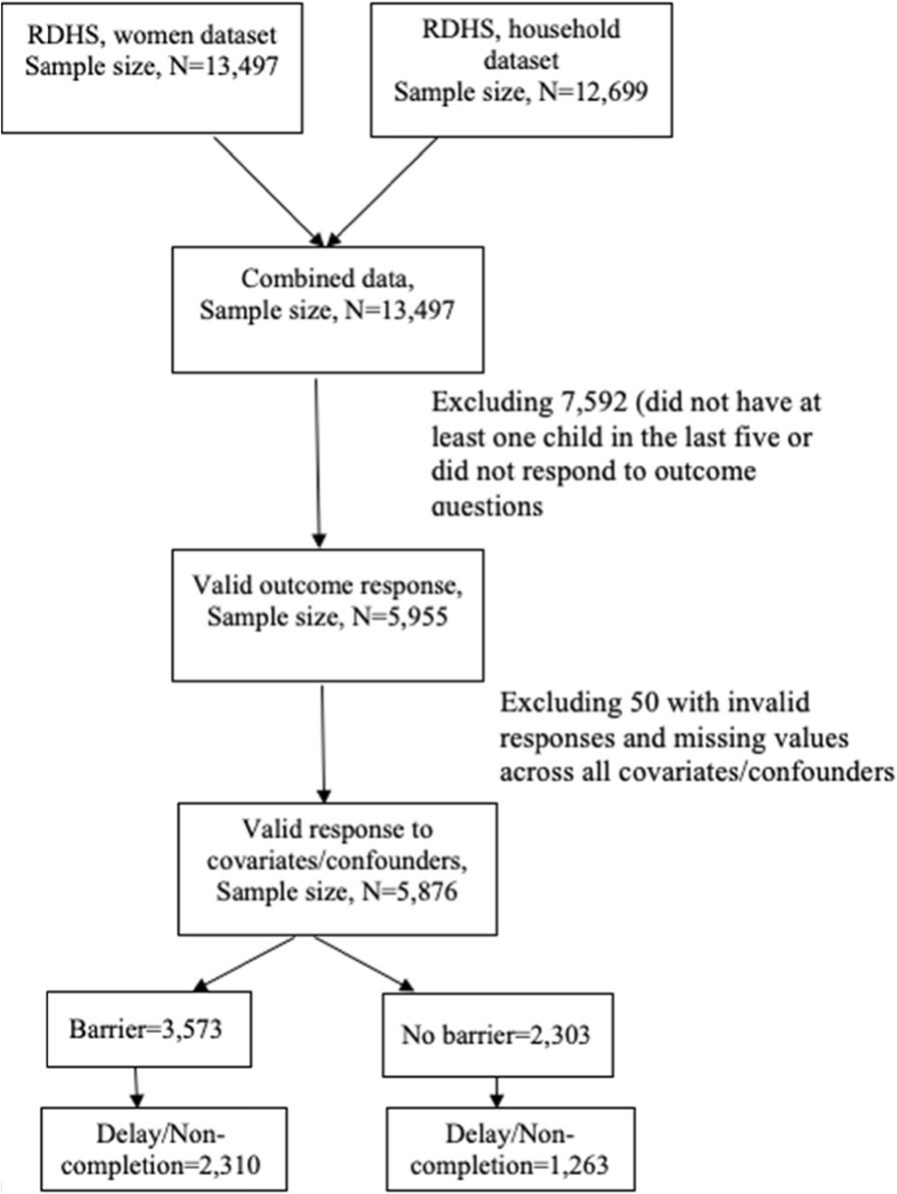
Flow chart of the analytic sample selection using women and household’s data from 2015 Rwanda Demographic and Health Survey (RDHS)

This study included women aged 15–49 years who had a child in the last 5 years preceding the survey and responded to antenatal care visits (ANC) and barriers to care questions. Women with missing values or invalid responses such as “don’t know,” “refused” or “not stated” to main exposure, outcome and potential confounders were excluded. Of 13, 497 women who participated in the survey, 5876 met the inclusion criteria. Figure 1 provides more details on the inclusion and exclusion criteria and derivation of the final analytic sample.

### Study variables

#### Outcome and exposure

The primary outcome of the study was inadequate ANC visits which is defined as “no” if the woman had at least four ANC visits and had first ANC visit within 3 months and “yes” if the number of visits were less than four or/and the first ANC visit was delayed (> 3 months) [4]. The exposure of interest was perceived barriers to accessing health care. Perceived barriers to care questions were combined to generate the exposure of the analysis. The exposure was “yes” if a woman perceived the following barriers to accessing health care: distance to the health facility, getting money for treatment, not wanting to go alone and getting permission to go for treatment. The exposure was “no” if none of these issues was perceived as barriers to accessing health care. To validate our findings, we conducted a separate analysis for each exposure variable separately and estimated the adjusted and unadjusted results.

#### Potential confounders and interactions

Potential confounders and predictors were identified based on the literature review [16, 21, 24]. Demographic and socio-economic variables included age, residence, marital status, level of education, employment status, insurance coverage, and household wealth index. The analysis included pregnancy history variables such as whether the last born to the woman was “planned and wanted,” “unplanned but wanted later”, unplanned or unwanted.” The respondents also reported the number of living children and this variable was included as continuous in the model. Lastly, the model included variables related to the woman’s access to information via radio or television and interaction terms between wealth and age groups. Table 1 shows more details on the categories of variables considered in the model. Additional file 1: Table S1 also gives more information on the comparison of exposed and unexposed group.

**Table 1:** Unadjusted association between inadequate ANC visits and potentially important variables: 2014–15 Demographic and Health Survey data

Variables	Overall sample	Timely with 4 visits	Not timely/< 4 visits or both	
	n (%^a^)	n (%^a^)	n (%^a^)	*P*-value^a§^
Sample size(n)	5876	2245	3631	
Barriers to care				< 0.001
No	2303 (38.3)	982 (42.3)	1321 (35.8)	
Yes	3573 (61.3)	2245 (57.7)	2310 (64.2)	
Pregnancy status				< 0.001
Planned and wanted	3556 (60.8)	1523 (67.8)	2033 (56.5)	
Unplanned but wanted later	1566 (26.4)	503 (22.4)	1063 (28.8)	
Unplanned and unwanted	754 (12.9)	219 (9.8)	535 (14.7)	
Age (%)				< 0.001
15–24 years	1243 (21.4)	472 (22.0)	771 (21.1)	
25–34 years	313 (52.7)	1257 (55.2)	1875 (51.2)	
35+ years	1501 (25.8)	516 (22.8)	985 (27.7)	
Wealth group				0.379
Poor	2594 (45.0)	954 (43.7)	1640 (45.8)	
Middle class	1106 (19.8)	430 (20.2)	676 (19.6)	
Rich	2176 (35.3)	861 (36.2)	1315 (34.7)	
Education				< 0.001
No education/primary	5007 (86.4)	1849 (84.0)	3158 (87.9)	
Secondary or higher	869 (13.6)	396 (16.0)	473 (12.1)	
Residence				0.725
Rural	4575 (83.1)	1727 (82.8)	2848 (83.3)	
Urban	1301 (16.9)	518 (17.2)	783 (16.7)	
Marital status				< 0.001
Never in union	614 (10.3)	172 (7.7)	442 (11.8)	
Married or living with partner	4685 (80.0)	1875 (83.5)	2810 (77.8)	
Previously married	577 (9.7)	198 (8.7)	379 (10.3)	
Employment status				0.015
Not working	387 (6.4)	128 (5.3)	259 (7.0)	
Skilled	994 (15.5)	418 (16.8)	576 (14.7)	
Unskilled	4495 (78.1)	1699 (77.8)	2796 (78.3)	
Watch TV or listen to radio at least once a week				0.012
No	2422 (42.1)	866 (39.8)	1556 (43.4)	
Yes	3454 (57.9)	1379 (60.2)	2075 (56.6)	
Insurance coverage (%)				< 0.001
No	1598 (27.7)	498 (23.0)	1100 (30.7)	
Yes	4278 (72.3)	1747 (77.0)	2531 (69.3)	
Number of living children (mean, SD)	2.8 (1.6)	2.6 (0.04)	2.9 (1.8)	< 0.001

*SD* Standard Deviation

§ *P*-values estimated using *Thomas-Rao modification*

^a^ Weighted estimates (adjusting for sampling weight, strata and sampling unit)

### Statistical analysis

We conducted bivariate analysis using Thomas-Rao corrections to a chi-square test [25, 26] to assess the association between categorical variables and the outcome, and survey-weighted t-test for the continuous variable. Variables that were statistically significant in bivariate analyses at the α = 0.05, as well as the important variables from the literature regardless of significance in the bivariate analysis, were retained for the initial model. We used variance inflated factors (VIFs) to assess multicollinearity between variables with VIFs < 2 indicating no meaningful multicollinearity in the data [27]. The model building used a survey-weighted multivariable logistic regression model [28]. Backward elimination method based on AIC [29] was used to select the final model; preferring the model with a smaller AIC. ANOVA was used further to justify the selection of a model. We additionally checked interactions in the model that made sense from domain-specific knowledge (Additional file 2).

The area under the Receiver Operating Characteristic (ROC) curve (AUC/C-statistic) [30], as well as Archer-Lemeshow test [29], were calculated to assess the goodness of fit of the final model. For the final model, we reported the odds ratio and 95% confidence intervals. We also conducted sensitivity analyses using propensity score weighting and sub-classification [31]. Additional details on these methods are explained in the Additional file 3. All the analyses accounted for the complex sampling design using sample weights, primary sampling unit and strata variables [32, 33]. We performed all analyses in R 3.5.1 [34].

## Results

### Study sample characteristics

Of 5, 876 women included in the analysis, 53% (3132) were aged between 20 to 34 years old, and 45% (2594) were in low wealth index. Eight-five percent (5007) of the women in the analytic sample had only primary education or lower, and 76% (4495) were doing an unskilled job at the time of the interview. The average number of children per woman was 3 (Standard Deviation (SD): 1.8). The prevalence of inadequate ANC visits was 62% (3631), and 61% (3573) of women reported having barriers to accessing health care (Table 1).

### Association between perceived barriers to health care, inadequate ANC visits and other potential variables

Table 1 also shows the survey-weighted association between inadequate ANC visits and other variables. Overall, 64% (2375) of women who perceived to have barriers to care had inadequate ANC visits. There was no multicollinearity between variables considered in the analysis (all VIFs< 2). Additional file 1: Table S1 presents the balance between the exposed and unexposed group. Table 2 presents the unadjusted and adjusted model findings. In the unadjusted analysis, women who reported having barriers to accessing health care were more likely to have inadequate ANC visits (Odds Ratio (OR): 1.31, 95% Confidence Interval (CI): 1.16, 1.49).

**Table 2:** Estimates from logistic regression assessing the relationship between perceived barriers to care and inadequate ANC visits: 2014–15 Demographic and Health Survey data

	Unadjusted relationship		Adjusted relationship	
Variables	OR^c^	95%CI^c^	OR ^c^	(95%CI) ^c^
Barriers to care				
No	Reference		Reference	
Yes	1. 31	(1.16, 1.49) ^a^	1.14	(0.99, 1.31)
Pregnancy status				
Planned and wanted	Reference		Reference	
Unplanned but wanted later	1.55	(1.36,1.76) ^a^	1.36	(1.18,1.56) ^a^
Unplanned and unwanted	1.80	(1.50,2.15) ^a^	1.29	(1.06,1.57) ^b^
Age				
15–24 years	Reference		Reference	
25–34 years	0.97	(0.85,1.10)	0.95	(0.82,1.10)
35+ years	1.26	(1.08,1.48)	0.96	(0.76,1.20)
Wealth group				
Poor	Reference		Reference	
Middle class	0.93	(0.79,1.09)	1.02	(0.87,1.20)
Rich	0.91	(0.80,1.05)	1.09	(0.94,1.27)
Education				
No education/primary	Reference		Reference	
Secondary or higher	0.72	(0.60,0.86) ^a^	0.74	(0.62, 0.90) ^a^
Residence				
Rural	Reference			
Urban	0.97	(0.81,1.16)	–	
Marital status				
Never in union	Reference		Reference	
Married or living with partner	0.61	(0.54,0.84) ^a^	0.59	(0.49, 0.72) ^a^
Previously married	0.77	(0.62,0.96) ^b^	0.71	(0.54, 0.94) ^b^
Employment status				
Not working	Reference		Reference	
Skilled	0.67	(0.54,0.84) ^a^	0.71	(0.56, 0.88) ^a^
Unskilled	0.77	(0.62,0.96) ^b^	0.68	(0.54, 0.85) ^a^
Watch TV or listen radio at least once a week				
No	Reference			
Yes	0.86	(0.77,0.97) ^b^	–	
Insurance coverage				
No	Reference		Reference	
Yes	0.67	(0.58,0.78) ^a^	0.73	(0.63, 0.85) ^a^
Number of living Children	1.11	(1.08, 0.14) ^a^	1.12	(1.07,1.17) ^a^

*OR* Odds ratio, *CI* Confidence interval

^a^ statistically significant at 1% level of significance,

^b^ significant at 5% level of significance

^c^ All estimates (OR, CI) are weighted using sampling weights, sampling unit and strata available in the 2014–15 DHS data

The direction of the relationship was the same in the adjusted model (OR: 1.14; 95%CI: 0.99, 1.31). Also, the adjusted model showed that having unplanned pregnancy and increased number of children born to a woman significantly contributed to inadequate ANC visits. In contrast, the following factors were protective from inadequate ANC visits: having secondary or higher education, having a partner or previously married, having a skilled or unskilled job and having an insurance coverage (Table 2). The model with interaction terms showed that wealthier and older women are less likely to have inadequate ANC visits compared to younger aged women (Additional file 2: Table S2). However, the inclusion of important interaction terms did not change the results; therefore, we considered the parsimonious model as the final model. The Archer-Lemeshow [35] goodness of fit test indicated that there was no evidence of lack-of-fit for the final model (*p* = 0.39) and The survey-weighted-receiver-operating curve [36] showed moderate discrimination (AUC = 0.61).

The analysis by individual variable used to create the perceived barriers to accessing health care exposure showed similar association across all variables except for the permission to go to the health facility (OR:0.94, 95% CI: 0.65, 1.39). Women who reported distance, money for treatment or not wanting to go alone to the health facility as a major issue were more likely to have inadequate ANC visits (Table 3).

**Table 3:** Relationship between perceived barriers to care variables and inadequate ANC visits: 2014–15 demographic and health survey data

	Timely with 4 visits	Not timely/< 4 visits or both		Unadjusted relationship	Adjusted relationship
Barriers to care variables	n (%^a^)	n (%^a^)	*P*-value^a^	OR (95% CI) ^a^	OR (95% CI) ^b^
Sample size (n)	2245	3631			
Do not want to go alone to the health facility					
No	1909 (85.1)	3019 (83.1)	0.056	Reference	
Yes	335 (14.9)	612 (16.9)		1.14 (0.96, 1.34)	1.10 (0.92,1.30)
Permission					
No	2193 (97.7)	3553 (97.9)	0.822	Reference	
Yes	51 (2.3)	78 (2.1)		1.02 (0.69,1.49)	0.94 (0.65, 1.39)
Distance to health facility					
No	1792 (79.8)	2794 (76.9)	0.011	Reference	
Yes	453 (20.2)	837 (23.1)		1.15 (0.99, 1.34)	1.12 (0.96,1.30)
Money for treatment					
No	1183 (52.7)	1617 (44.5)	< 0.001	Reference	
Yes	1062 (47.3)	2014 (55.5)		1.32 (1.17, 1.49)	1.13 (0.98,1.29)

*OR* Odds Ratios, *CI* Confidence Interval

a All percentages, OR and CI are weighted using sampling weights available in the 2014–15 DHS data

^b^ Adjusted for pregnancy status, wealth group, residence, education, marital status, age, parity, insurance coverage, employment status

### Sensitivity analysis using propensity score methods

The sensitivity analyses using propensity score methods [31] showed similar magnitude and direction of the relationship as the main analysis results (Additional file 3: Table S3). Additional file 3: Table S3 shows that across all propensity score methods, women who reported having barriers to care were more likely to have inadequate ANC visits. More details on the propensity score methods and findings are presented in the Additional file 3.

## Discussion

In this study, we found that almost two-thirds of women with perceived barriers to accessing health care had inadequate ANC visits. Women who reported having barriers to care were more likely to have inadequate ANC visits, however, the association was not statistically significant. The results were consistent in the sensitivity analysis using propensity score methods and when we investigated the relationship between each perceived barrier and the outcome separately. Although we cannot draw causal conclusions, our results are similar to other studies that assessed barriers to ANC services utilization in SSA. A survey conducted in Nigeria found that non-users of ANC services were women who had problems of getting money for treatment, and those who lived far from the health facility and had issues of transport facilities [37]. Similarly, to these studies, the current study showed that women who had reported distance to health facility and money for treatment to be barriers were more likely to have inadequate ANC visits.

Several studies conducted in SSA found that women who could not get permission to go to a health facility were less likely to use ANC services [19, 37, 38]. We noted that women who reported permission to go to a health facility as a perceived barrier to health care were less likely to have inadequate ANC visits. This finding is not consistent with what we anticipated and found for other barrier factors because of the small sample size. Only very few women reported permission to be a barrier to health care which might have led to unstable estimates and change of direction in the relationship.

### Demographic and socio-economic status factors

Marital status was significantly associated with inadequate ANC visits, which is consistent with prior evidence [21]. This is in part linked to women’s availability to visit a health facility. Women who live with their partners might have more support and time in their schedule to seek care compared to single mothers. We found women’s education level to be a significant predictor of inadequate ANC visits. Generally, educated women are aware of the benefits of regular checkup during pregnancy and more informed about the timing of each ANC visits. Other studies [39, 40] showed that women do not attend ANC because they are not familiar or do not understand the value of ANC especially those who did not experience any complications in their prior pregnancy or those whose pregnancy is their first (primigravida).

Contrary to previous findings on ANC utilization [22, 41, 42], age and wealth group were not significantly associated with the outcome in our analysis. However, we kept these variables in the model based on a priori knowledge [41] and included the interaction term between these two variables in the model for sensitivity analysis. The findings showed that wealthier and older women are less likely to have inadequate ANC visits compared to younger aged and poorer women. This relationship is explained by the fact that the wealthier and older women group might have more experience with motherhood and are likely more informed about pregnancy compared to the poorer and younger counterparts. Our results on these two factors are consistent with another study conducted in Rwanda that did not find age and wealth group to be significant predictors of the timing of the first ANC visit [21].

Although other studies showed that women’s poverty is linked to ANC services utilization [37, 42], this might not be the case in Rwanda due to the community-based health insurance (CBHI) scheme, commonly known as *mituelle de Sante*. We found that 72% of women were insured through CBHI which gave women access to ANC services at the lowest price or no cost. Though CBHI, Rwanda has universal health coverage [43] that allows women to access healthcare at an affordable cost; however, there is still a small percentage of women who are uninsured mainly due to financial constraints and are more likely to not attend ANC visits. The government of Rwanda in collaboration with the World Bank has started a program that provides financial support and employment to the households in the lowest wealth index and those who cannot afford to pay CBHI premiums [44]. This program will likely help this group of the population to have access to care, and future research should investigate the impact of this program on health services utilization including ANC among women beneficiaries across the country.

Employment status was a protective factor from inadequate ANC visits which is consistent with previous findings [21]. Women who are employed might be more informed and have financial autonomy to access health care compared to unemployed women as many other studies have reported [4, 21, 42].

### Pregnancy status

Previous studies have reported pregnancy and parity to be a significant barrier for ANC services utilization [4, 45], and we also observed a similar relationship in the current analysis. Women’s feelings about unplanned pregnancy might influence their health care seeking behavior which in turn can increase risks of pregnancy complications or mortality [46]. Furthermore, women who have more children face time constraints which affect their health care services utilization and affordability of health care services. Strengthening family planning programs in Rwanda could help families to have planned pregnancy and hence the desired number of children which in turn could improve ANC services utilization and boost families’ economic status. Our findings suggest further efforts in a comprehensive reproductive and sexual health education focusing on the efficient use of contraceptives to prevent risks related to unplanned pregnancy and birth spacing for women in Rwanda.

### Study strengths and limitations

This study had a number of strengths. The study used a representative sample which allows generalizability of the findings to other population in similar resources settings as Rwanda. Additionally, we conducted several sensitivity analyses such as propensity score methods to validate our results, and we investigated each barrier separately to estimate individual association with the outcome. We found consistent findings across all these types of confounding control approaches suggesting the robustness of the study findings.

Our study has some limitations that need to be acknowledged. First, owing to using secondary data, there are some factors that have been reported in the previous studies as important predictors of ANC services utilization that were not collected in 2014–15 RDHS. Those variables include quality of care, waiting time at a health facility, expertise of health care providers and culture practices [16, 39]. Failure to control for these variables might explain the moderate discrimination (60%) that we obtained in our analysis. Second, our study might have suffered from social desirability bias. For instance, women might have underreported barriers to care which might be the reason why we observed a lower number of women who reported permission to be a major problem. Further, current study might have been subject to recall bias in case women who had birth close to the time of the survey might have remembered information more prominently than women who had birth back in time. Lastly, our study is a cross-sectional study, and we cannot draw causal inferences based on our findings. However, the consistency of results obtained using different methods gives us more confidence in our estimates.

## Conclusion

The study showed that women who perceived to have barriers to accessing care were more likely to have a delayed first ANC visit and not complete at least four recommended visits; however, the association was borderline statistically significant. We observed other behavior, socio-economic and demographic factors also significantly to contribute to inadequate ANC visits. Implementation of programs improving access to health care such as decentralization of ANC services to health post level could improve ANC services utilization. Health posts are health facilities that are closest to patients’ home. Decentralizing ANC services to this level of care would reduce the distance that women have to travel to the health centers. These findings also suggest that achieving adequate ANC visits requires sustained, coordinated effort across many sectors. Rwanda Ministry of Health should prioritize programs aiming at improving health care-seeking behavior, pregnancy planning, and interventions that will enhance women’s knowledge about ANC. Timely and more frequent ANC visit will contribute to reducing maternal and neonatal mortality and morbidity.

## Supplementary information

**Additional file 1: **Comparison of exposed and unexposed group. **Table S1.** Sample characteristics by exposure variable (perceived barriers to health care): 2015 Demographic and Health Survey data.

**Additional file 2: **Adjusted logistic regression including an interaction term between wealth group and age. **Table S2.** Estimates from logistic regression assessing the relationship between perceived barriers to health care and inadequate ANC visits including an interaction between age and wealth group: 2015 Demographic and Health Survey data.

**Additional file 3: **Sensitivity analysis using propensity score methods. **Table S3.** Propensity score findings of the relationship between perceived barriers to care and delayed and non-completion of antenatal care visits: 2015 Demographic and health survey (DHS) data.

## Data Availability

The datasets used during the current study are available from the worldwide DHS website (https://dhsprogram.com/data/available-datasets.cfm) and registration is required for access to data.

## Abbreviations

AIC: Akaike Information riterion
ANC: Antenatal Care
AUC: Area Under the Curve
BC: British Columbia
CBHI: Community-Based Health Insurance
CI: Confidence Interval
FANC: Focused Antenatal Care
OR: Odds Ratio
RDHS: Rwanda Demographic and Health Survey
ROC: Receiver Operating Characteristic
SD: Standard Deviation
SPPH: School of Population and Public Health
SSA: sub-Saharan Africa
VIFs: Variance Inflated Factors
WHO: World Health Organization
