# Supplementary material for: Are perceived barriers to accessing health care associated with inadequate antenatal care visits among women of reproductive age in Rwanda?
Source: BMC Pregnancy Childbirth. 2020 Feb 10;20:88. doi: 10.1186/s12884-020-2775-8 (PMC7011379; doi:10.1186/s12884-020-2775-8)
Supplement: Supplementary file 1 — Additional file 1: Comparison of exposed and unexposed group. Table S1. Sample characteristics by exposure variable (perceived barriers to health care): 2015 Demographic and Health Survey data. [file 12884_2020_2775_MOESM1_ESM.docx]

**Additional file**

**Additional file 1—Comparison of exposed and unexposed group**

**Additional file 1: Table S1: Sample characteristics by exposure variable (perceived barriers to health care):** 2**015 Demographic and Health Survey data**

|  | Perceived barriers to health care | |  |
| --- | --- | --- | --- |
| Variables | **No**  **n (%^†^)** | **Yes**  **n (%^†^)** | **SMD^†^** |
| Sample size | 2,303 | 3,573 |  |
| Inadequate ANC visits _*_ |  |  | 0.13 |
| No | 982(42.0) | 1,263(35.6) |  |
| Yes | 1,321(58.0) | 2,310 (64.4) |  |
| Pregnancy status |  |  | 0.16 |
| Planned and wanted | 1,488(64.7) | 2,068 (58.3) |  |
| Unplanned but wanted later | 587(25.2) | 979 (27.1) |  |
| Unplanned and unwanted | 228(10.1) | 526 (14.6) |  |
| Age (%) ^*^ |  |  | 0.10 |
| 15-24 years | 519 (23.4) | 724 (20.2) |  |
| 25-34 years | 1,245 (53.0) | 1,887 (52.5) |  |
| 35+ years | 539(23.6) | 962 (27.3) |  |
| Wealth group (%) |  |  | 0.79 |
| Poor | 550 (24.3) | 2,044 (57.8) |  |
| Middle class | 432 (20.6) | 674 (19.3) |  |
| Rich | 1,321 (55.1) | 855 (22.9) |  |
| Education (%) |  |  | 0.42 |
| No education/primary | 1,722(77.2) | 3,285 (92.2) |  |
| Secondary or higher | 581 (22.8) | 288 (7.8) |  |
| Residence (%) |  |  | 0.36 |
| Rural | 1,547(74.5) | 3,028 (88.4) |  |
| Urban | 756(25.5) | 545(11.6) |  |
| Marital status (%) ^*^ |  |  | 0.22 |
| Never in union | 204 (9.1) | 410 (11.0) |  |
| Married or living with partner | 1,955(84.7) | 2,730 (77.1) |  |
| Previously married | 144(6.1) | 433 (11.9) |  |
| Employment status (%) |  |  | 0.47 |
| Not working | 194(8.1) | 193 (5.3) |  |
| Skilled | 638 (25.5) | 356 (9.3) |  |
| Unskilled | 1,471(66.4) | 3,024 (85.4) |  |
| Watch TV or listen to radio at least once a week (%) |  |  | 0.60 |
| No | 545 (24.8) | 1877 (52.8) |  |
| Yes | 1,758 (75.2) | 1,696 (47.2) |  |
| Insurance coverage (%) |  |  | 0.61 |
| No | 267 (12.2) | 1331 (37.4) |  |
| Yes | 2,036 (87.8) | 2,242 (62.6) |  |
| Parity (mean, SD) | 2.6(1.7) | 2.9(1.8) | 0.34 |

*SMD: Standard Mean Difference for unmatched data*

† *All percentages and SMD are weighted using sampling weights*

** Balanced covariates at SMD=0.25*
