# Supplementary material for: Are perceived barriers to accessing health care associated with inadequate antenatal care visits among women of reproductive age in Rwanda?
Source: BMC Pregnancy Childbirth. 2020 Feb 10;20:88. doi: 10.1186/s12884-020-2775-8 (PMC7011379; doi:10.1186/s12884-020-2775-8)
Supplement: Supplementary file 2 — Additional file 2: Adjusted logistic regression including an interaction term between wealth group and age. Table S2. Estimates from logistic regression assessing the relationship between perceived barriers to health care and inadequate ANC visits including an interaction between age and wealth group: 2015 Demographic and Health Survey data. [file 12884_2020_2775_MOESM2_ESM.docx]

**Additional file 2: Adjusted logistic regression including an interaction term between wealth group and age**

We conducted a survey-weighted multivariate logistic regression including an interaction between wealth group and age. The table below shows the findings with the interaction term.

**Additional file 2 Table S2: Estimates from logistic regression assessing the relationship between perceived barriers to health care and inadequate ANC visits including an interaction between age and wealth group: 2015 Demographic and Health Survey data**

| Variables | OR^†a^ | 95%CI^†a^ |
| --- | --- | --- |
| Barriers to care |  |  |
| No | Reference |  |
| Yes | 1.13 | (0.99, 1.31) |
| Wealth group by age |  |  |
| 15-24 years (Middle class vs poor) | 0.92 | (0.66, 1.28) |
| 15-24 years (Rich vs poor) | 1.39 | (1.04, 1.84) ^*^ |
| 25-34 years (Middle class vs poor) | 0.99 | (0.79, 1.22) |
| 25-34 years (Rich vs poor) | 1.04 | (0.85, 1.26) |
| 35+ years (Middle class vs poor) | 1.19 | (0.86, 1.63) |
| 35+ years (Rich vs poor) | 1.02 | (0.79, 1.31) |
| Age by wealth group |  |  |
| Poor (25-34 vs 15-24 years) | 1.03 | (0.85, 1.25) |
| Poor (35+ vs 15-24 years) | 1.01 | (0.76, 1.35) |
| Middle class (25-34 vs 15-24 years) | 1.10 | (0.79, 1.52) |
| Middle class (35+ vs 15-24 years) | 1.30 | (0.87, 1.92) |
| Rich (25-34 vs 15-24 years) | 0.77 | (0.60, 0.99) ^*^ |
| Rich (35+ vs 15-24 years) | 0.75 | (0.54,1.02) |

*OR: Odds ratio, CI: Confidence interval*

*^*^ significant at 5% level of significance*

**^†^** *All estimates (OR, CI) are weighted using sampling weights, sampling unit and strata available in the 2015 DHS data*

^a^ *estimate adjusted for unintended pregnancy, education, marital status, employment status, insurance coverage and parity*
