# Supplementary material for: Are perceived barriers to accessing health care associated with inadequate antenatal care visits among women of reproductive age in Rwanda?
Source: BMC Pregnancy Childbirth. 2020 Feb 10;20:88. doi: 10.1186/s12884-020-2775-8 (PMC7011379; doi:10.1186/s12884-020-2775-8)
Supplement: Supplementary file 3 — Additional file 3: Sensitivity analysis using propensity score methods. Table S3. Propensity score findings of the relationship between perceived barriers to care and delayed and non-completion of antenatal care visits: 2015 Demographic and health survey (DHS) data. [file 12884_2020_2775_MOESM3_ESM.docx]

**Additional file 3—Sensitivity analysis using propensity score methods**

We conducted two types of propensity score methods, and we estimated a propensity score for having barriers to care for each woman using unweighted multivariate logistic regression model including the following variables: unintended pregnancy, wealth group, education, residence, employment, insurance coverage, and parity.

We first conducted the stratified propensity score using three subclasses (determined based on small sample size). Stratum 1 had imbalances in 5 covariates. These variables were included in the final model to estimate the association between the exposure variable and the outcome. Furthermore, we carried out a propensity score weighting. We used an inverse probability of treatment weighting (IPTW) technique suitable for survey data to estimate the association between exposure and outcome. IPTW weights were multiplied by the survey weights so that the multiplied weights represent the pseudo-population which is an unconfounded version of the target population. There were some moderately extreme weights (weights > 20), and therefore we decided to do 1% truncation. Logistic regression model accounting for the calculated weights were used to estimate the association between exposure and outcome. Web-Table C.1 below shows the findings from each method.

**Additional file 3 Table S3: Propensity score findings of the relationship between perceived barriers to care and delayed and non-completion of antenatal care visits: 2015 Demographic and health survey (DHS) data**

| Analysis type | Unadjusted association  (OR) ^†^ | 95% CI^†^ | weight mean (min-max) | Overall association (OR) | 95% CI |
| --- | --- | --- | --- | --- | --- |
| Propensity score sub- classification^*‡^ | 1.31 | (1.16, 1.49) |  | 1.14 | (0.97, 1.34) |
| Propensity score weighting truncated |  |  | 0.96(0.08,6.38) | 1.12 | (0.92, 1.31) |

*OR: Odds Ratios, CI: Confidence interval*

**Balance checked at standardized mean difference (SMD)=0.25*

*OR and CI are survey-weighted*

**^‡^** *Three subclasses were considered in the analysis*

^†:^ *Estimates before adjusting for propensity scores and unbalanced variables*
